# The identity of *Callicarpa minutiflora* Y. Y. Qian (Lamiaceae) and taxonomic synonym of *C. longifolia* Lamarck

**DOI:** 10.3897/phytokeys.75.10704

**Published:** 2016-11-29

**Authors:** Zhonghui Ma, Zhiwei Su

**Affiliations:** 1 Agricultural College, Guangxi University, Nanning, Guangxi, 530004,China; 2 Guangxi Key Laboratory of Marine Environmental Science, Guangxi Academy of Sciences, Nanning, Guangxi, 530007, China

**Keywords:** Callicarpa minutiflora, Callicarpa longifolia, Lamiaceae

## Abstract

Although the specific epithet of *Callicarpa minutiflora* Y. Y. Qian has been revised for many times, during the study of the genus *Callicarpa*, we find that *Callicarpa minutiflora* Y. Y. Qian is identical to *Callicarpa longifolia* Lamarck by a series of morphologic characters. In order to avoid more confusion, here *Callicarpa minutiflora* Y. Y. Qian is reduced as a synonym of *Callicarpa longifolia* Lamarck.

## Introduction

The genus *Callicarpa* L. is currently treated as incertae sedis after being transferred from the family Verbenaceae to Lamiaceae (Harley et al. 2004, Bramley 2009, 2013, Olmstead 2010, 2012, Ma et al. 2015). This genus comprises approximately 140 species mainly distributed in temperate, subtropical and tropical Asia, America, Australia and the Pacific Islands (Harley et al. 2004, Bramley 2009, 2013, Ma et al. 2015), with 48 species and 13 varieties occurring in China (Chen and Gilbert 1994). Although some regional revisions (especially in Southeast Asia) of *Callicarpa* have been completed (Munir 1982, Leeratiwong et al. 2009, Bramley 2009, 2013), this genus is still taxonomically problematic due to lack of field investigations and specimens available for study in some species.

Among the Chinese species with 4-angled branchlets and a conspicuous interpetiolar transverse ridge resembling a stipule scar, *Callicarpa acutifolia* Chang, *Callicarpa longifolia* Lamarck, *Callicarpa longissima* (Hemsley) Merrill and *Callicarpa minutiflora* Y. Y. Qian are always troublesome to distinguish between each other. The last species *Callicarpa minutiflora* Y. Y. Qian was described based on the type collection (Y.Y. Qian 1800) from Jiangcheng County, Yunnan Province, China and no other collections could be found. However, this specific epithet is a later homonym of earlier *Callicarpa minutiflora* Rusby and therefore illegitimate. Although this problem was overlooked during preparation of *Flora of China* (Chen and Gilbert 1994), recently Duan and Zhang (2014) discovered this illegitimate name when they updated the *Flora of China* online version and proposed a new replaced name *Callicarpa tenuiflora* Li Bing Zhang & Yi F. Duan. Unfortunately, this new name is also a later homonym of legitimate earlier *Callicarpa tenuiflora* Champ. ex Benth. characterised by very short petiole or subsessile and cordate leaf base which is considered as a synonym of *Callicarpa rubella* Lindley (Leeratiwong et al. 2009). Again, Zhang (2014) proposed *Callicarpa qianyiyongii* Li Bing Zhang as a new replaced name for the later homonym *Callicarpa tenuiflora* Li Bing Zhang & Yi F. Duan.

**Figure 1.**
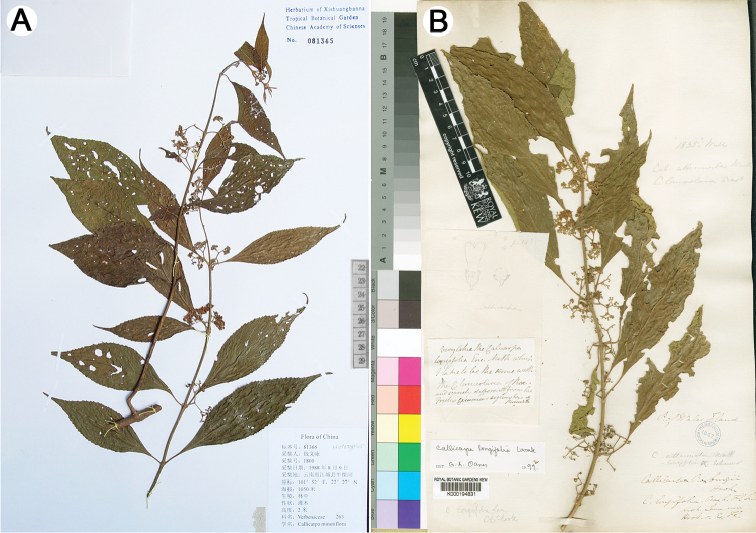
Type of *Callicarpa minutiflora* Y. Y. Qian and *Callicarpa longifolia* Lamarck.

**Figure 2.**
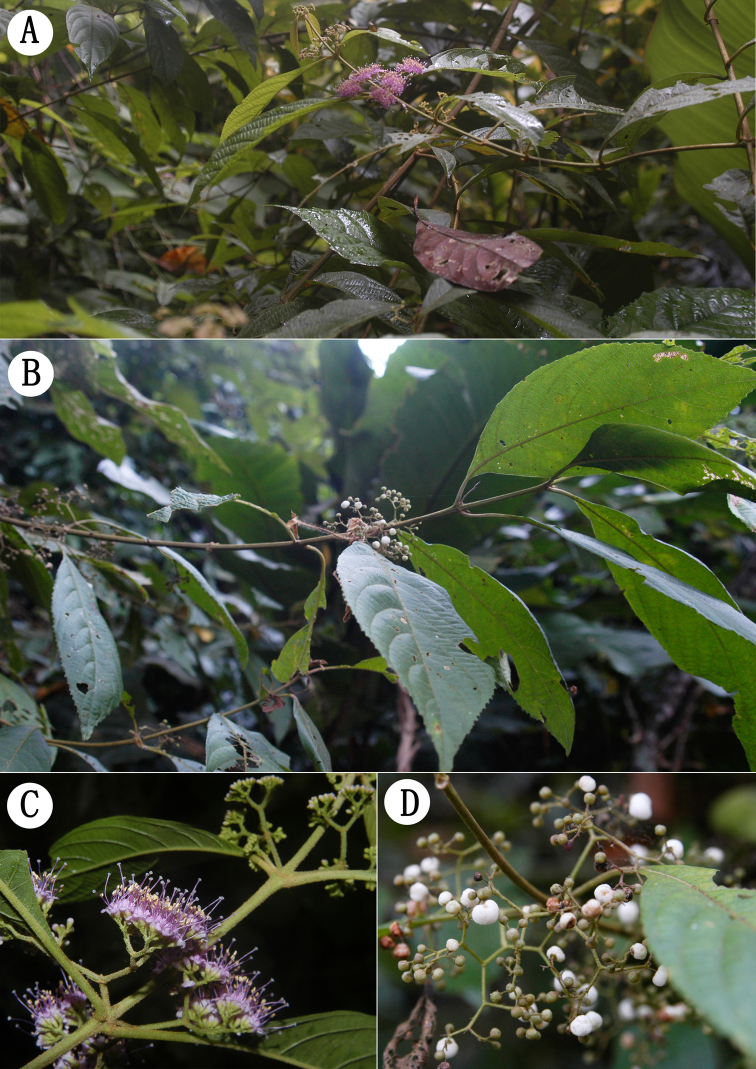
Field images of *Callicarpa longifolia* Lamarck. **A, C** branch with flowers (Z.H. Ma ZHM0154 IBSC) **B, D** branch with white fruits (Z.H. Ma ZHM0117 IBSC).

In the protologue, Qian (1991) stated that *Callicarpa minutiflora* Y. Y. Qian was similar to *Callicarpa acutifolia* Chang with 4-angled, grooved branchlets and a transverse scar in the node, but could be distinguished from the latter by its elliptic leaf blade, shorter peduncle compared with petiole, as well as densely tomentellous calyx, corolla and ovary (Figure 1). However, after examination of type specimens and original descriptions, we find that *Callicarpa minutiflora* Y. Y. Qian is identical to *Callicarpa longifolia* Lamarck by its 4-angled branchlets, conspicuous interpetiolar ridge, elliptic leaf blade and by covering of dense gray pubescent and yellowish sessile glands on branchlets, petioles, both sides of leaf blade (especially dense on the midrib and venation), peduncles, ovary and outer surface of calyx and corolla (Figures 1, 2), which obviously differs from other *Callicarpa* species. Additionally, the plant of *Callicarpa minutiflora* Y. Y. Qian is ca. 2 m tall; petiole slender, 1–2.5 cm; leaf blade 10–16 × 4–7 cm, base cuneate, apex acuminate; peduncle 0.6–1.5 cm; calyx 0.7–1 mm, subtruncate; corolla pale pink to lilac, 2–2.5 mm; fruit globose; flowering in Jul.–Aug. and fruiting in Aug.–Oct.; distributed in forests, at an elevation of about 1100 m (Qian 1991, Chen and Gilbert 1994). Although the color of mature fruit is still unknown, these characters above are also perfectly consistent with or in the range of variation of those of *Callicarpa longifolia* (Figures 1, 2).

In order to avoid more confusion, it is essential to reduce *Callicarpa minutiflora* Y. Y. Qian as a synonym of *Callicarpa longifolia* Lamarck.

## Taxonomic treatment

### Callicarpa longifolia

Taxon classificationPlantaeLamialesLamiaceae

Lamarck (1785: 563)

Callicarpa minutiflora Y.Y. Qian (1991: 121), *nom. illeg.*, non Callicarpa minutifloraRusby (1927: 339); Callicarpa tenuiflora Li Bing Zhang & Yi F. Duan (2014: 278), *nom. illeg.*, non Callicarpa tenuiflora Champ. ex Benth. (1853: 135); Callicarpa qianyiyongii Li Bing Zhang (2014: 57), **syn. nov.** Type: China. Yunnan, Jiangcheng, forests, 1050 m, 9 Aug 1988, Y.Y. Qian 1800 (holotype, HITBC!; isotype, SYS!).

#### Type.

Malaysia, Malacca, Sonnerat, s.n. (holotype P-LA); Malaysia, Hooker, J.D., s.n. (syntype K, K000194831, microfiche!).

#### Distribution.

China, Pakistan, India, Bhutan, Bangladesh, South-East Asia through to New Guinea, Australia.

#### Ecology.

In the edge of secondary forest and disturbed areas, such as roadsides, streamside, or open patches in primary forest. Alt. 0–1300 m (Leeratiwong et al. 2009, Bramley 2009, 2013, Chang 1951).

#### Other specimens examined.

**China.** Guangdong: Zhaoqing, H.G. Ye 45 (IBSC!); Gaoyao, C. Huang 161917 (IBSC!); Yangshan, L. Deng 1313 (PE!); Luoding, Z.H. Ma ZHM003, ZHM 005 (IBSC!). Guangxi: Lingle, Z.Q. Zhang 10547 (IBSC!); Cangwu, S.Q. Chen 9936 (IBSC!); Ningming, Z.H. Ma ZHM0154 (IBSC!). Hainan: Wanning, F.W. Xing 5563 (IBSC!); Z.X. Li 4758 (IBSC!); Baoting, K.Z. Hou 72820 (IBSC!, PE!); Anding, Z. Huang 35683 (IBSC!, PE!); Baisha, X.Q. Liu 25599 (IBSC!, PE!); Ganen, X.Q. Liu 4875 (IBSC!, SYS!); Sanya, Z.X. Li 2611 (IBSC!); Ledong, X.Q. Liu 27118 (IBSC!); Dongfang, S.Q. Chen 11225 (IBSC!). Jiangxi: Jinggangshan, J. Xiong 3181 (PE!). Yunnan: Pingbian, H.T. Tsai 61314 (IBSC!), 61385 (PE!), P.Y. Mao 3033 (IBSC!); Mengla, C.W. Wang 80038 (PE!); Hekou, Anonymous 1741 (PE!); Jinghong, G.D. Tao 17611 (PE!), P.W. Xie 10-145 (IBSC!), Z.H. Ma ZHM0117 (IBSC!); Cangyuan, Y.H. Li 012620 (SYS!). Hongkong: S.Y. Hu 10193 (PE!); Tsiang Ying 609 (SYS!). **Vietnam.** China-Vietnam Expedition 1211 (IBSC!). **India.** U. Singh 81 (IBSC!). **Indonesia**. Johns, R.J. 9851 (K!), J.A. Lorzing 13463 (K!). **Australia.** Drinkel, C. 2 (K). **Papua**. L.J. Brass 1013 (K!), S. Isles & A. Vinas 34486 (K!). **Malaysia.** R. Schlechter 13818 (K!), M. Jacobs 8477 (K!). **Sumatra.** P. Buwalda 6661 (K!), N. Walter & M.B, Catherine 640, 987 (K!), R.J. Morley & M. K. Kardin Morley306 (K!).

## Supplementary Material

XML Treatment for Callicarpa longifolia
